# The protective role of Sirt1 in vascular tissue: its relationship to vascular aging and atherosclerosis

**DOI:** 10.18632/aging.101068

**Published:** 2016-10-15

**Authors:** Munehiro Kitada, Yoshio Ogura, Daisuke Koya

**Affiliations:** ^1^ Department of Diabetology and Endocrinology, Kanazawa Medical University, Uchinada, Ishikawa, Japan; ^2^ Division of Anticipatory Molecular Food Science and Technology, Medical Research Institute, Kanazawa Medical University, Uchinada, Ishikawa, Japan

**Keywords:** Sirt1, endothelial cell, macrophage, aging, atherosclerosis

## Abstract

Cardiovascular disease (CVD) due to atherosclerosis is the main cause of death in both the elderly and patients with metabolic diseases, including diabetes. Aging processes contribute to the pathogenesis of atherosclerosis. Calorie restriction (CR) is recognized as a dietary intervention for promoting longevity and delaying age-related diseases, including atherosclerosis. Sirt1, an NAD^+^-dependent deacetylase, is considered an anti-aging molecule and is induced during CR. Sirt1 deacetylates target proteins and is linked to cellular metabolism, the redox state and survival pathways. Sirt1 expression/activation is decreased in vascular tissue undergoing senescence. Sirt1 deficiency in endothelial cells (ECs), vascular smooth muscle cells (VSMCs) and monocytes/macrophages contributes to increased oxidative stress, inflammation, foam cell formation, senescences impaired nitric oxide production and autophagy, thereby promoting vascular aging and atherosclerosis. Endothelial dysfunction, activation of monocytes/macrophages, and the functional and phenotypical plasticity of VSMCs are critically implicated in the pathogenesis of atherosclerosis through multiple mechanisms. Therefore, the activation of Sirt1 in vascular tissue, which includes ECs, monocytes/macrophages and VSMCs, may be a new therapeutic strategy against atherosclerosis and the increasing resistance to the metabolic disorder-related causal factors of CVD. In this review, we discuss the protective role of Sirt1 in the pathophysiology of vascular aging and atherosclerosis.

## INTRODUCTION

Cardiovascular disease (CVD) due to atherosclerosis is the most frequent cause of death in both the elderly and patients with metabolic disorders such as diabetes mellitus [1, 2], metabolic syndrome [3], hypertension and dyslipidemia. Aging is a systemic process that affects all organs, and age-related disruptions in cellular homeostasis lead to declines in organ function and impaired responses to physiological stress. In addition, aging is closely linked to metabolic disorders including insulin resistance, diabetes, hypertension and dys-lipidemia, and the impairment of metabolic health accelerates cellular and tissue aging. Although aging is not thought to be the specific cause, it is an independent risk factor for CVD because both the incidence and the prevalence of CVD increase dramatically after the age of 50 years for men and women [4]. Therefore, elucidating the mechanisms underlying aging may lead to breakthroughs in the treatment of atherosclerosis. Several mechanisms of atherosclerosis that are related to the aging process have been proposed [5]. The free radical theory of aging suggests that the damage to cellular macromolecules induced by the accumulation of reactive oxygen species (ROS) is a primary driving force of aging and atherosclerosis [6]. In addition, low-grade inflammation associated with the activation of the transcription factor nuclear factor-κB (NF-κB) in the vascular wall is related to the pathogenesis of vascular aging, atherosclerosis [7, 8] and oxidative stress. Furthermore, autophagy is a bulk degradation process that is involved in the clearance of damaged proteins and organelles and is highly conserved from yeast to mammals [9]. Autophagy works to maintain cellular homeostasis under various conditions of intracellular stress. Therefore, autophagy deficiencies can lead to inabilities to counteract age-related cellular damage and may therefore accelerate aging, whereas enhanced appropriate autophagy activity may prolong a healthy life span. Furthermore, the impairment of autophagy may be involved in age-related diseases, including atherosclerosis.

Calorie restriction (CR) without malnutrition promotes longevity and slows aging [10]. A possible mechanism by which CR exerts such beneficial effects involves the actions of sirtuins, particularly Sirt1 [11]. Sirt1 is associated with the regulation of a wide variety of cellular processes, including metabolism, the inhibition of apoptosis, mitochondrial biogenesis, anti-oxidative stress responses, anti-inflammatory pathways and the induction of autophagy [12]. Therefore, Sirt1 may exert protective effects against vascular aging and atherosclerosis. Additionally, Sirt1 modulates the generation of endothelial nitric oxide (NO), a protective factor for endothelial cells that exerts an anti-atherosclerotic effect [13]. Endothelial dysfunction, the activation of monocytes/macrophages and the functio-nal and phenotypical plasticity of vascular smooth muscle cells (VSMCs) are critically implicated in the pathogenesis of atherosclerosis through multiple mechanisms. In this review, we discuss regarding the role of Sirt1 activity in the pathogenesis of vascular aging and atherosclerosis, particularly focusing on the beneficial effects of Sirt1 against cellular senescence, inflammation, oxidative stress, autophagy deficiency and impairment of NO production.

### Characteristics of Sirt1

CR leads to a variety of beneficial effects, including extending lifespan and delaying the onset of age-related diseases such as CVD, neurodegenerative disorders and diabetes. CR has also been established as an experimental anti-aging paradigm [14, 15]. Sir2 (silent information regulator 2), an NAD^+^-dependent deacetylase, was initially identified from studies of aging in yeast [16] and has been established as one of the molecules through which CR extends lifespan or delays the pathogenesis of aging-related diseases. Homologs of Sir2 in higher eukaryotic organisms are known as sirtuins. Sirt1, which is the sirtuin most closely related to Sir2, is one of the seven known mammalian sirtuins. Sirt1 functions as a class III histone deacetylase and binds to NAD^+^ and acetyllysine residues within its protein targets to generate lysine, 2′-O-acetyl-Adenosine diphosphate (ADP)-ribose, and nicotinamide as enzymatic products. Nicotinamide acts as a negative-feedback inhibitor of Sirt1. Although Sirt1 exists in the cytoplasm, it is found predominantly in the nucleus, where a large fraction is associated with euchromatin [11].

Mammalian Sirt1 promotes chromatin silencing and transcriptional repression through histone deacetylation. Upon its recruitment to chromatin, Sirt1 can directly deacetylate histone (H) 4 lysine 16 (H4K16), H3 lysine 9 (H3K9), H3 lysine 14 (H3K14) and H1 lysine 26 (H1K26) [16, 17]. These deacetylations promote the hypoacetylation of nucleosomal histones and reduce the transcription of the corresponding DNA. Furthermore, because histone acetylation and methylation are often coordinately regulated, deacetylation by Sirt1 may also promote alterations in histone methylation. Sirt1 may enhance histone H4 lysine 20 monomethylation (H4K20me) and H3 lysine 9 trimethylation (H3K9me3) and may reduce H3 lysine 79 dimethylation (H3K79me2) [18].

In addition, more than a dozen non-histone proteins, including transcription factors, transcriptional coregulatory proteins and several enzymes, serve as substrates for Sirt1. Furthermore, a metabolite produced by the activity of Sirt1 can exert a beneficial effect in preventing metabolic derangement. Pazienza et al, showed that macroH2A1.1, which is a variant of histone H2A, binds O-acetyl ADP ribose (a small metabolite produced from the reaction catalyzed by Sirt1) with high affinity and protects hepatocytes against lipid accumulation, resulting in the suppression of non-alcoholic steatohepatitis [19]. Therefore, the activation of Sirt1 can exert many physiological effects, including reduced apoptosis, enhanced mitochondrial biogenesis, the inhibition of inflammation, the regulation of glucose and lipid metabolism, the regulation of the circadian rhythm, the induction of autophagy and adaptations to cellular stresses such as hypoxia, endoplasmic reticulum (ER) stress and oxidative stress [11, 12, 20].

The expression and activity of Sirt1 in many mammalian cell types (including vascular endothelial cells) are regulated by several cellular stresses and transcriptional, post-transcriptional and post-translational modifications, including microRNA (miR) or phosphorylation, methylation, sumoylation and nitrosylation. Transcription factors such as E2F transcription factor 1(E2F-1), Forkhead box Os (FOXOs), Hypermethylated In Cancer 1 (HIC1), p53, and cMYC have been identified to modulate Sirt1 expression under oxidative stress, DNA damage conditions, and nutrient deprivation. E2F-1 directly binds to the Sirt1 promoter at a consensus site and appears to regulate the basal expression levels of Sirt1 [21], while high levels of Sirt1 lead to a negative-feedback loop in which E2F-1 activity is inhibited by Sirt1-mediated deacetylation. Nemoto et al. showed that under starvation conditions, FOXO3 stimulates Sirt1 expression, which requires an interaction with p53 [22]. Furthermore, Sirt1 transcription is upregulated by MYC, which binds at the Sirt1 promoter region, and by signal transducers and activators of transcription 5 (STAT5), which binds at positions in response to BCR-ABL oncogenic stress [23]. By contrast, the Sirt1 promotor has the two binding sites for the tumor suppressor HIC1, and direct binding of HIC1 to these binding sites represses Sirt1 transcription [24]. In addition, two functional p53 binding sites, which normally repress Sirt1 expression under normal nutrient conditions, have been identified [22]. Sirt1 expression is also regulated at the post-transcriptional level by Hu antigen R (HuR) [25]. It has been demonstrated that HuR, a ubiquitously expressed RNA binding protein, associates with the 3′ UTR of Sirt1 mRNA under physiological conditions and helps to stabilize the transcript. This interaction results in increased Sirt1 mRNA stability and subsequently elevated protein levels. Conversely, the HuR-Sirt1 mRNA complex is disrupted upon oxidative stress, which leads to decreased mRNA stability and therefore decreased Sirt1 protein levels.

The microRNAs are important regulatory factors of Sirt1. Several miRs such as miR-34a, -181a, -9, -146, -143, -132, -34c and -217 directly regulate the expression of Sirt1 in many tissues and cells [26]. Among these miRs, miR-34a and -217 have been implicated in the expression of Sirt1 in vascular endothelial cells. Ito et al. reported that aging endothelial cells expressed high levels of miR-34a and low levels of Sirt1 protein, and overexpression of miR-34a decreased Sirt1 protein levels and increased acetylated p53 levels in endothelial cells, suggesting that miR-34a regulates endothelial senescence in part through Sirt1 [27]. Menghini et al. also showed that miR-217 induces a premature senescence-like phenotype and leads to impaired angiogenesis via inhibition of Sirt1 and modulation of FOXO1 and endothelial NO synthase (eNOS) acetylation. Conversely, inhibition of miR-217 in older endothelial cells ultimately reduces senescence and increases the angiogenic activity via an increase in Sirt1. Additionally, miR-217 is expressed in human atherosclerotic lesions and is negatively correlated with Sirt1 expression and the FOXO1 acetylation status [28].

In the context of the cellular stress response, two proteins have been identified to regulate Sirt1 activity both positively and negatively through complex formation. The active regulator of Sirt1 (AROS) was identified as a direct regulator of Sirt1 [29]. A negative regulator of Sirt1, Deleted in Breast Cancer-1 (DBC-1), which binds directly to the catalytic domain of Sirt1, also has been identified [30]. Both factors represent the first endogenous direct regulators of Sirt1 function. In addition, Sirt1 activity is subjected to regulation by other post-translational modifications such as phosphorylation, sumoylation and methylation in response to stress signaling and cell cycle changes. Using a mass spectrometry approach, 13 phosphoryla-tion sites were identified in Sirt1 [31]. Seven of these phosphorylation sites, including Ser27 and Ser47, are located in the N-terminal region of Sirt1 and are phosphorylated by c-Jun N-terminal kinase (JNK), resulting in the enhancement of Sirt1 nuclear localization. However, mammalian target of rapamycin (mTOR)-dependent phosphorylation of Ser47 alone results in the inhibition of Sirt1 deacetylase activity [32]. Additionally, cyclin-dependent kinase 5 (CDK5)-mediated hyperphosphorylation of Sirt1 at Ser47 con-tributes to the development of endothelial senescence and atherosclerosis [33]. Six phosphorylation sites in the C-terminal region, including Thr530 and Ser540, are potential substrates of cyclin B/cyclin-dependent kinase 1 (CDK) complexes, which is associated with normal cell progression. There are four phosphorylation sites in the N- and C-terminal extensions (Ser154, Ser649, Ser651, and Ser683) in mice, and two of these sites have been described in human Sirt1 at the corresponding amino acids Ser659 and Ser661 [34]. Ser659 and Ser661 within human Sirt1 lie within a region that is referred to as the essential for Sirt1 activity motif, and CK2 phosphorylates these sites to contribute to the control of Sirt1 catalytic activity [34].

In addition, sumoylation is another important post-translational modification for the regulation of Sirt1 activation. Sirt1 activity is increased by sumoylation at K734 on the C-terminal end of Sirt1, which is repressed by sentrin-specific peptidase 1 (SENP1) [35]. Sumoylation enhances Sirt1 catalytic activity during ischemic preconditioning in the mouse heart [36]. However, it is unclear how modification by SUMO stimulates the catalytic activity of Sirt1. Several possible mechanisms by which sumoylation of Sirt1 increases its activity have been proposed. There is a possibility that sumoylation of Sirt1 may modify CK2 phosphorylation, and subnuclear relocalization of sumoylated Sirt1 may affect accessibility to substrates or produce allosteric effects of the sumoylation.

Sirt1 is also targeted by methylation. The methyl-transferase Set7/9 both interacts with and methylates Sirt1 at Lys233, Lys235, Lys236, and Lys238 on the N-terminal extension, which interacts with Set7/9 [37]. Although it is unclear whether methylation directly affects Sirt1 deacetylase activity, the interaction of Set7/9 with Sirt1 disrupts the binding of Sirt1 with p53, resulting in increased p53 acetylation.

### Role of Sirt1 in vascular aging and atherosclerosis

Endothelial dysfunction is characterized by impaired endothelium-dependent vasorelaxation and represents an early step in the pathogenesis of atherosclerosis [38]. The known mechanisms of atherosclerosis related to endothelial dysfunction include impaired NO production by eNOS and defects in its signaling pathway, increased oxidative stress, inflammation and impaired autophagy [5]. In addition to endothelial dysfunction, activation and infiltration of immune cells such as monocytes/macrophages as well as foam cell formation in the vascular walls are critical steps in atherogenesis [39], which is also related to inflam-mation and oxidative stress. Previous reports also show that autophagy in monocytes/macrophages plays a crucial role in the pathogenesis of atherosclerosis by reducing oxidative stress and inflammation and suppressing foam cell formation. Additionally, adipose and liver tissues are either directly or indirectly involved in the pathogenesis of vascular aging and atherosclerosis, particularly through the systemic inflammation and oxidative stress associated with insulin resistance [40]. Furthermore, the functional and phenotypic plasticity of VSMCs (which are associated with oxidative stress, inflammation and impaired autophagy) plays an important role in the progression of atherosclerosis. Vascular aging is closely related to vascular injury due to endothelial dysfunction by impairment of eNOS function, inflammation, oxidative stress and dysregulation of autophagy in vascular cells including not only endothelial cells but also monocytes/macrophages and VSMCs [41, 42]. Therefore, a common mechanism and therapeutic target may exist during the process of vascular aging and atherosclerosis.

**Figure 1:**
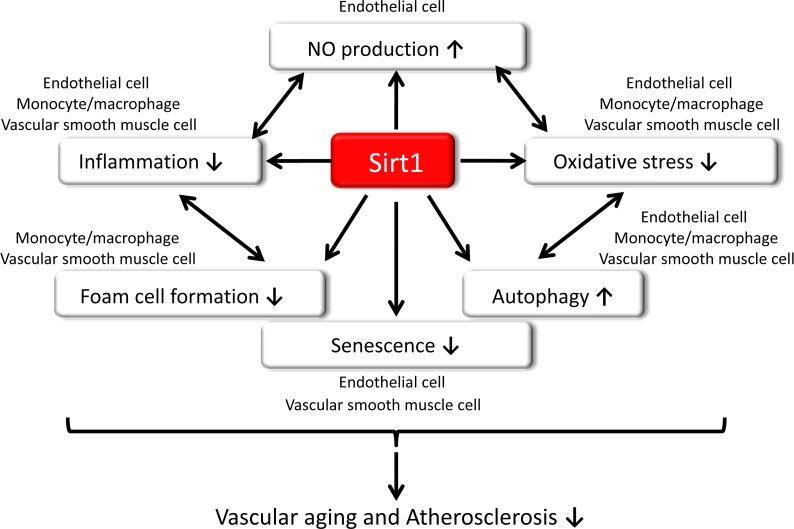
Role of Sirt1 in vascular aging and atherosclerosis Sirt1 may increase nitric oxide (NO) production, decrease inflammation, reduce oxidative stress, induce autophagy, and prevent senescence, resulting in the suppression of vascular aging and atherosclerosis.

What is the role of Sirt1 in vascular aging and atherosclerosis? Essentially, Sirt1 is highly expressed in endothelial cells and regulates angiogenic function during vascular growth [43] and vascular function, including NO production from eNOS. Additionally, reduced Sirt1 expression facilitates the manifestation of senescence in endothelial cells [44, 45, 46]. Donato et al. previously reported that Sirt1 expression is reduced in endothelial cells obtained from arteries of older human adults (64 ± 1 years) compared to those from younger adults (25 ± 1 years), and reduced endothelial Sirt1 expression with aging may be related to endothelial dysfunction through the impairment of NO production from eNOS [47]. Recently, Bai et al. demonstrated the molecular mechanisms underlying the preventive effect of endothelial senescence and vascular aging [48]. Sirt1 prevents endothelial senescence by enhancing Liver kinase B1 (LKB1) degradation by its deacetylation, which causes an interaction with HECT and RLD domain containing E3 ubiquitin protein ligase 2(HERC2), a giant scaffolding protein and E3 ubiquitin ligase. In senescent endothelial cells or aged arteries, loss of Sirt1 expression or function results in an increased nuclear accumulation of acetylated LKB1, leading to irreversible structural alterations of the blood vessel wall, adverse arterial remodeling and vascular stiffness. Additionally, Thompson et al. reported that Sirt1 expression in young and old human VSMCs isolated from human arteries from donors ranging in age from 12 to 82 years show an inverse correlation with age [49]. They also showed that a significant difference in Sirt1 levels from human VSMCs isolated from aged and occluded arteries with atherosclerotic lesions compared with non-occluded sections of the same artery. Thus, reduced expression or activity of Sirt1 is observed in vascular cells, which is associated with aging and may contribute to the natural development of vascular dysfunction. Furthermore, Badi et al. reported that miR-34a was highly expressed in aortas isolated from older mice, and downregulation of Sirt1 by miR-34a in VSMCs promotes senescence and inflammation [50]. By contrast, it is unclear whether Sirt1 expression in circulating monocytes or macrophages in the vascular wall reduces with aging. However, previous reports have shown that reduced Sirt1 expression in isolated monocytes or mononuclear cells from human subjects may be related to systemic or possibly vascular wall inflammation, which contributes to atherosclerosis. These data indicate that reduced Sirt1 in vascular cells and monocytes/macrophages contribute to the pathogenesis of atherosclerosis and is associated with vascular aging. Therefore, the protective effects of Sirt1 on vascular aging and atherosclerosis are proposed to act by increasing NO production, HERC2-mediated degradation of LKB1, anti-inflammatory activity, anti-oxidative stress and the induction of autophagy (Figure 1).

### Sirt1 modulates eNOS and NO production

**Figure 2:**
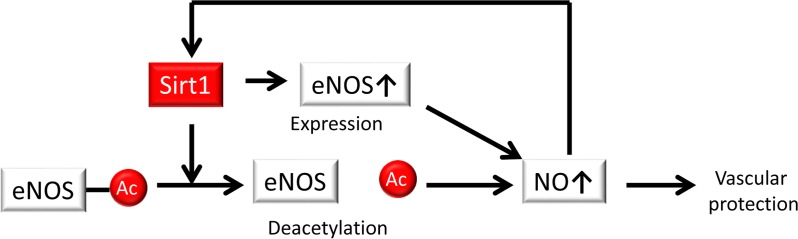
Sirt1 regulates NO production Sirt1 regulates endothelial NO synthase (eNOS) at both the transcriptional and post-transcriptional levels via deacetylation, resulting in the generation of NO for vascular protection. NO also positively regulates Sirt1.

It is well established that endogenous NO generated from eNOS plays a crucial role in maintaining vascular function and homeostasis, which facilitates vascular tone, leukocyte adhesion, smooth muscle cell proliferation and migration, and platelet aggregation. Previous studies have shown that endogenous NO serves as an anti-atherosclerotic and anti-aging factor and that Sirt1 in endothelial cells regulates NO production. Donato et al. demonstrated a role for decreased Sirt1 expression and eNOS activity in age-associated endothelial dysfunction in mice [47]. They demonstrated that protein expression levels of Sirt1 and phosphorylated eNOS at serine 1177 in the aortas of 30-month-old B6D2F1 mice were lower than those in 5- to 7-month-old mice and that acetylated eNOS levels were markedly higher in 30-month-old mice compared to the younger mice, whereas total eNOS levels did not differ. Acetylcholine (ACh)-induced peak endothelium-dependent dilatation was also lower in isolated femoral arteries from aging mice. In addition, Mattagajasingh et al. showed that Sirt1 promotes endothelium-dependent vasodilation by eNOS activation-mediated increases in NO production in calorie-restricted mice [13]. Sirt1 and eNOS colocalize and coprecipitate in endothelial cells, and CR-induced Sirt1 leads to the activation of eNOS due to deacetylation of lysines 496 and 506 in the calmodulin-binding domain of eNOS [13]. By contrast, suppression of Sirt1 in the endothelium of arteries inhibits endothelium-dependent vasodilation and decreases endogenous NO [13]. Furthermore, studies of mice with genetically altered Sirt1 (Sirt1-Tg mice) revealed a protective role of Sirt1 in endothelial cells [51, 52, 53]. Zhang et al. reported that the high-fat diet-induced impairment of endothelium-dependent vasorelaxation in ApoE^−/−^ mice was improved com-pared with that of wild-type littermates in the Sirt1-Tg mice and was accompanied by an upregulation in aortic eNOS expression [52]. Sirt1-Tg/ApoE^−/−^ mice had fewer atherosclerotic lesions compared with ApoE^−/−^ controls, whereas the blood lipid and glucose levels were unaffected. By contrast, Stein showed that Sirt1 does not influence endothelium-dependent vascular function in ApoE^−/−^ mice even though p-eNOS (Ser1177) expression in the aortic ring was increased in Sirt1^+/+^/ApoE^−/−^ mice, but Sirt1 prevented superoxide production in endothelial cells and reduced the expression of inflammatory adhesion molecules by suppressing NF-κB signaling. However, difference of the acetylation levels of eNOS in the aortic ring was not shown in Sirt1^+/+^/ApoE^−/−^ and Sirt1^+/−^/ApoE^−/−^ mice [53]. Thus, Sirt1 is implicated in the regulation of eNOS at both the transcriptional and post-transcriptional levels to generate NO, leading to vascular protection (Figure 2).

Additionally, NO may positively regulate Sirt1 expression. Nisoli et al. demonstrated that Sirt1 expression is regulated by NO in white adipose tissue (WAT) and white adipocytes [54]. CR leads to increasing Sirt1 expression in the white adipose tissue of wild-type mice, while eNOS knockout mice showed no change in Sirt1 expression. Furthermore, Sirt1 expression was significantly higher in cultured white adipocytes exposed to either NO donors such as (Z)-1- E2-(2-aminoethyl)-N-(2-ammonioethyl) amino) diazen-1-ium-1,2 diolate (DETA-NO) and S-nitrosoacetyl penicillamine (SNAP) or a cGMP analog (8 Br-cGMP) compared to untreated cells and was lower in the WAT of eNOS^−/−^ mice when compared with wild-type animals [54]. However, it is unclear whether NO produced from eNOS positively regulates Sirt1 expression in endothelial cells. Ota et al. reported that a phospho-diesterase (PDE) 3 inhibitor (cilostazol) prevented the oxidative stress that occurs from premature endothelial senescence by NO-generated Sirt1 upregulation via the activation of eNOS, which is phosphorylated by cyclic adenosine monophosphate (cAMP)/protein kinase A (PKA) [55]. In addition, Ota and colleagues reported that statins phosphorylated Akt at Ser473, which led to the increased expression of eNOS and Sirt1 in cultured endothelial cells, and the inhibition of NO by treatment with either NG-nitro-L-arginine methyl ester (L-NAME) or siRNA against eNOS reduced the expression of Sirt1, ultimately resulting in vascular protection [56]. Thus, a positive-feedback loop involving the eNOS-NO-Sirt1 axis exists to protect against endothelial senescence and atherosclerosis (Figure 2).

### Sirt1 suppresses atherosclerosis inflammation

The inflammatory process is a critical mechanism underlying the initiation and progression of aging and age-related diseases including atherosclerosis [57]. Both endothelial cell dysfunction and activated immune cells such as monocytes/macrophages are involved in vascular low-grade inflammatory processes [39, 58]. The chronic activation of the nuclear factor-kappa B (NF-κB) signaling pathway in endothelial cells and activated immune cells play a central role in the inflammatory processes that occur during vascular aging and atherosclerosis. Chronic NF-κB activation leads to the overexpression of inflammation-related genes such as cellular adhesion molecules (e.g., intercellular adhesion molecule-1 (ICAM-1), vascular adhesion molecule-1 (VCAM-1), monocyte chemo-attractant protein-1 (MCP-1), inducible nitric oxide synthase (iNOS) and cyclooxygenase-2 (COX-2)) and pro-inflammatory cytokines (e.g., tumor necrosis factor-α (TNFα), interleukin-6 (IL-6) and interleukin-1β (IL-1β)), thus conferring an inflammatory phenotype that contributes to endothelial dysfunction. In addition to endothelial dysfunction and immune cell activation, adipose and liver tissues influence the generation of increased systemic inflammation, which is also associated with atherosclerotic pathogenesis.

**Figure 3:**
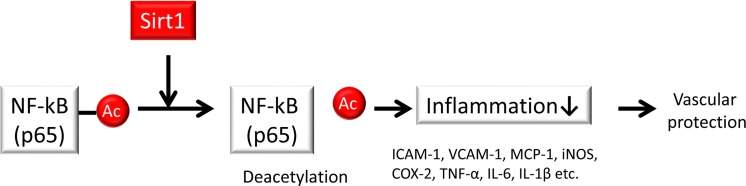
Sirt1 regulates inflammation Sirt1 improves inflammation by deacetylating the nuclear factor-κB (NF-κB) p65 subunit to inhibit the expression of inflammation-related genes such as intercellular adhesion molecule-1 (ICAM-1), vascular adhesion molecule-1 (VCAM-1), monocyte chemoattractant protein-1 (MCP-1), inducible NO synthase (iNOS), and cyclooxygenase-2 (COX-2) as well as pro-inflammatory cytokines such as tumor necrosis factor-α (TNF-α), interleukin-6 (IL-6) and interleukin-1β (IL-1β) in endothelial cells and monocytes/macrophages.

What is the mechanism by which Sirt1 negatively regulates inflammation? The acetylation of several lysines of the p65 subunit of NF-κB may contribute to the potential regulatory function of NF-κB [59]. Among these acetylated lysine residues in NF-κB p65, acetylation at K310 may promote superior transcriptional activity while also acting as a substrate for Sirt1 [60]. Therefore, Sirt1 may negatively regulate NF-κB activity by deacetylating K310 on the p65 subunit (Figure 3). Reduced levels of Sirt1 lead to the upregulation of acetylated NF-κB, resulting in increased inflammatory responses in adipocytes [61], monocytes/macrophages [62] [63], myeloid cells [64], endothelial cells [53] and microglia [65] in several experimental animal and cell culture models. In those studies, Sirt1 upregulation (via either chemical activators of Sirt1 or overexpression of the Sirt1 gene) reduced inflammation through the deacetylation of NF-κB.

The relationship among Sirt1, inflammation, vascular aging and atherosclerosis has been demonstrated and contributes to metabolic disorders such as insulin resistance. A previous report has shown that Sirt1 expression is decreased in monocytes from patients with insulin resistance [66]. We also demonstrated that 7 weeks of CR in obese male patients upregulated Sirt1 expression in their peripheral blood mononuclear cells, was associated with decreased expression of inflammation-related markers in the circulation and was negatively correlated with homeostasis model assessment as an index of insulin resistance (HOMA-IR), an insulin resistance index [67]. In addition, Breitenstein et al. demonstrated that Sirt1 mRNA expression is decreased in peripheral blood monocytes isolated from patients either with angiographically confirmed coronary artery disease and/or presenting with acute coronary syndrome compared with mono-cytes isolated from healthy subjects [68], and the expression of IL-6 was increased in patients with impaired Sirt1 expression. Furthermore, Li et al. showed that Sirt1 expression in peripheral blood mononuclear cells was negatively correlated with the levels of plasma inflammatory cytokines and chemo-kines, including IL-6 and TNF-α, in patients with type 2 diabetes and coronary artery disease [69]. These data suggest that Sirt1 expression in monocytes or mononuclear cells negatively regulates inflammation and is involved in atherosclerosis. A study using myeloid-specific Sirt1 knockout mice revealed that animals challenged with a high-fat diet displayed high levels of activated macrophages in the liver and adipose tissues, predisposing them to the development of systemic insulin resistance and metabolic abnormalities [64] that are possibly related to atherosclerosis. Thus, Sirt1 plays an important role in regulating inflammation in the settings of vascular aging and atherosclerosis.

### Sirt1 suppresses oxidative stress in the vasculature

Oxidative stress has been recognized as one of the causative factors in vascular aging and atherosclerosis. Oxidative stress is induced by an imbalance between the overproduction of ROS from pro-oxidative enzymes (e.g., xanthine oxidase, nicotinamide adenine dinucleo-tide phosphate (NADPH) oxidase, uncoupled eNOS or enzymes of mitochondrial respiration) and the inactivation of anti-oxidative molecules (e.g., copper/zinc-superoxide dismutase (Cu/Zn-SOD), manganese (Mn)-SOD, extracellular SOD). Inflammation and oxidative stress are closely linked, thus giving rise to a vicious cycle in the process of aging and atherosclerosis [42].

**Figure 4:**
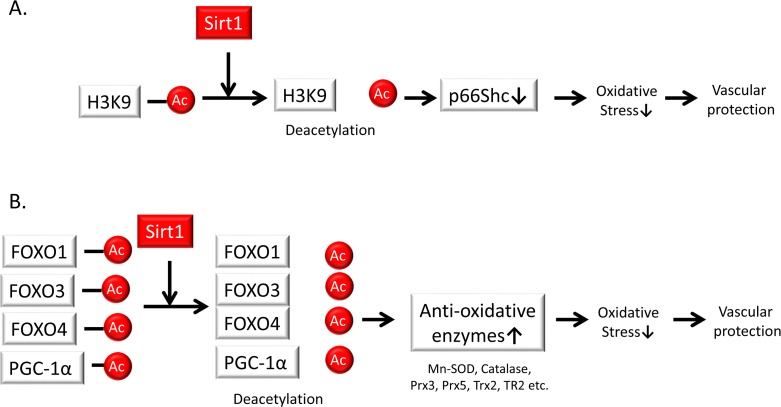
Sirt1 regulates oxidative stress (A) Sirt1 represses p66Shc transcription through deacetylation of the histone H3 lysine 9 promoter. This crosstalk between p66Shc and Sirt1 serves as a mechanism for preventing vascular diseases based on anti-oxidative stress responses. (B) Sirt1 regulates cellular oxidative stress through the induction of anti-oxidative enzymes such as a manganese superoxide dismutase (Mn-SOD), catalase, peroxiredoxins 3 and 5 (Prx3, Prx5), thioredoxin 2 (Trx2), and thioredoxin reductase 2 (TR2) via the deacetylation and activation of Forkhead box O (FOXO) 1, 3, and 4 transcription factors as well as peroxisome proliferator-activatedreceptor gamma coactivator-1 (PGC-1α) in endothelial cells.

Mitochondria are one of the major sources of ROS and therefore play a crucial role in aging and aging-related diseases, including atherosclerosis. The adapter protein p66Shc has the ability to directly stimulate mitochondrial ROS generation through its oxido-reductase activity. Indeed, p66Shc-deficient mice have increased resistance to oxidative stress and prolonged lifespans; p66Shc-deficient mice are protected against age-related and hyperglycemia-induced endothelial dysfunction and exhibit less atherosclerosis when fed a high-fat diet. Previous studies have also shown that Sirt1 can repress p66Shc transcription through the deacetylation of H3K9 at its promoter [70]. The crosstalk between p66Shc and Sirt1 prevents vascular diseases by contributing to anti-oxidative stress responses and inhibiting endothelial senescence (Figure 4A).

Sirt1 has been shown to regulate cellular oxidative stress through the induction of anti-oxidative enzymes, Mn-SOD, catalase, peroxiredoxins 3 and 5 (Prx3, Prx5), thioredoxin 2 (Trx2), and thioredoxin reductase 2 (TR2) via the deacetylation and activation of the FOXO3 transcription factor and Peroxisome proliferator-activated receptor gamma coactivator 1-alpha (PGC-1α) in endothelial cells [71]. Alcendor et al. also reported that moderate overexpression of Sirt1 in mice protects their hearts from oxidative stress due to increased expression of catalase via FOXO1a [72]. FOXO4 is also acetylated by stimulation of hydrogen peroxide through acetyltransferases such as the related proteins p300 and cyclic AMP response element-binding protein (CBP); upon binding of p300 and CBP, FOXO4 is deacetylated by Sirt1 [73]. Another study reported that liver-specific Sirt1 deficiency caused an increase in ROS production, which disrupted the mTOR 2/Akt signaling pathway in other insulin-sensitive organs, resulting in insulin resistance [74]. In addition, oxidative stress and oxidized low-density lipoprotein (oxLDL) increases DNA damage in VSMCs in atherosclerosis through reduced Sirt1 expression. Gorenne et al. showed that endogenous Sirt1 is reduced in human atherosclerosis, particularly in VSMCs [75] Sirt1 deficiency reduces DNA repair and promotes the activation of DNA damage markers and senescence via reduced activation of the repair protein Nijmegen breakage syndrome-1 (NBS-1) due to decreased deacetylation. These results in VSMC apoptosis, reduced relative fibrous cap thickness, medial degeneration, aneurysm formation, and aortic dissection. Thus, the involvement of Sirt1 in the regulation of cellular oxidative stress may depend on the availability of its direct substrates (Figure 4B).

Chronic oxidative stress leads to the presence of high levels of ROS (e.g., superoxide (O_2_^−^) and hydrogen peroxide (H_2_O_2_)) in aged vessels, resulting in chronic activation of the redox-sensitive NF-κB transcription factor. Inflammation promotes further ROS generation resulting in a positive-feedback loop. Sirt1 can attenuate oxidative stress through multiple mechanisms as described above. However, oxidative stress from excess ROS and oxLDL also decreases the activity and expression of eNOS, leading to reduced NO levels and Sirt1 activity, which negatively modulate NF-κB activation. This interplay results in a vicious cycle of Sirt1 inactivation and oxidative stress/inflammation in vascular tissue, resulting in vascular aging and atherosclerosis. Therefore, Sirt1 may be a protective molecule that can break this cycle such that its activation is beneficial in suppressing vascular aging and atherosclerosis.

### Sirt1 suppresses foam cell formation in atherosclerosis plaques: relationship to oxidative stress, inflammation, cholesterol uptake and reverse transport in macrophages

**Figure 5:**
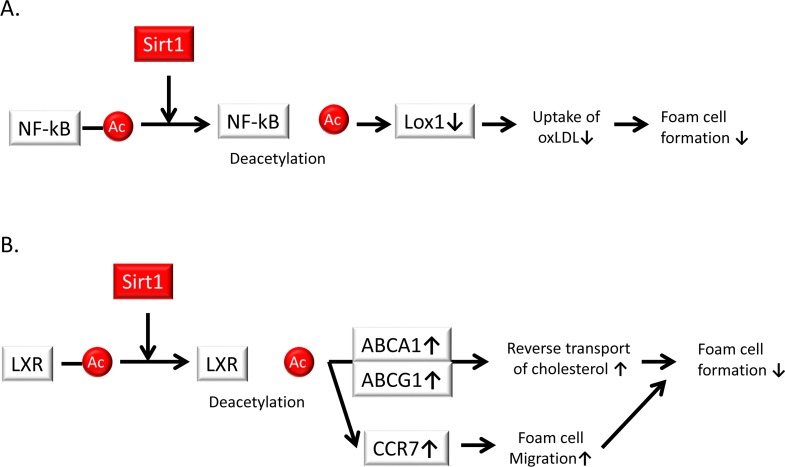
Sirt1 suppresses foam cell formation in atherosclerotic plaques (**A**) Sirt1 reduces the uptake of oxidized low-density lipoprotein (oxLDL) by diminishing the expression of lectin-like oxidized LDL receptor-1 *(Lox-1)* via the deacetylation and suppression of the NF-κB signaling pathway, leading to a reduction of foam cell formation. (**B**) Sirt1 interacts with the liver X receptor (LXR) and promotes its deacetylation and subsequent activation. Activation of LXR upregulates the expression of ATP-binding cassette sub-family A member (ABCA) 1 and ABC sub-family G member (ABCG) 1, which leads to the reverse transport of cholesterol and the suppression of foam cell formation and cholesterol loading in macrophages. The induction of C-C chemokine receptor type 7 (CCR7) contributes to foam cell migration, resulting in a reduction of foam cell formation.

The infiltration of monocyte-derived macrophages into the subendothelial space is a crucial step in atherogenesis and contributes to inflammation. The accumulation of cholesterol in macrophages and resultant formation of foam cells is induced by the uptake of modified LDL (mainly oxLDL) through scavenger receptors such as lectin-like oxLDL receptor 1 (Lox-1). Stein et al. demonstrated that Sirt1 protects against atherosclerosis by reducing macrophage foam cell formation in Sirt1^+/+^ApoE^−/−^ mice compared to that in Sirt1^+/−^ApoE^−/−^ mice. Peritoneal macrophages from Sirt1^+/−^ mice accumulate more oxLDL and thus promote foam cell formation. Additionally, bone marrow-restricted Sirt1 deletion confirmed that Sirt1 function in macrophages is sufficient to decrease atherogenesis. Moreover, Sirt1 reduces the uptake of oxLDL by diminishing the expression of Lox-1 via the deacetylation and suppression of the NF-κB signaling pathway [76] (Figure 5A).

Additionally, liver X receptors (LXRs) serve as a cholesterol sensor to protect against cholesterol overload. The activation of LXRs confers beneficial effects in lipid metabolism and CVD, including the stimulation of cholesterol efflux from cells to high-density lipoproteins (i.e., reverse cholesterol transport) through ATP-binding cassette (ABC) transporters, specifically ABC sub-family A (ABCA) 1 and ABC sub-family G member (ABCG) 1, which activates the conversion of cholesterol to bile acids in the liver to facilitate excretion. The impairment of reverse cholesterol transport may lead to excessive accumulation of cholesterol in cells and promote foam cell formation. Sirt1 interacts with LXRs and promotes its deacetylation (K432 in LXRα and K433 in LXRβ) and subsequent activation [77]. Therefore, the Sirt1-LXR-ABCA1/ABCG1 signaling pathway is involved in the regulation of cholesterol efflux and is inhibited during the initiation and progression of atherosclerosis, resulting in cholesterol uptake by macrophages and foam cell formation (Figure 5B). In addition, Zeng et al. showed that downregulation of the LXR signaling pathway during foam cell formation is mediated by the suppression of Sirt1 in a high-triglyceride, high-cholesterol environment. Activation of Sirt1 leads to the increased expression of LXRs and their corresponding target genes, including ABCA1 and ABCG1 as well as C-C chemokine receptor type 7 (CCR7), which is induced in foam cells and promotes foam cell migration from atherosclerotic plaques. Thus, Sirt1 may prevent the generation and progression of atherosclerosis by enhancing the LXR-ABCA1/ABCG1/CCR7 pathway to suppress foam cell formation [78] (Figure 5B).

### Autophagy in atherosclerosis

**Figure 6:**
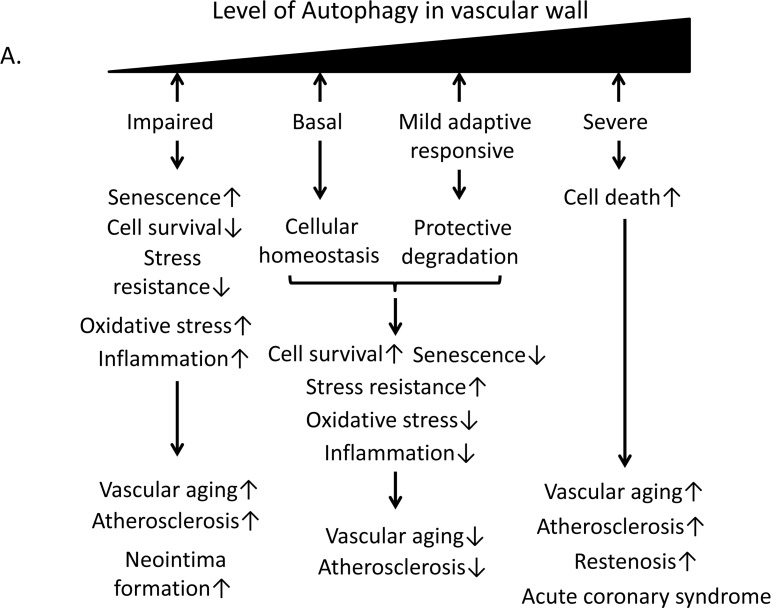

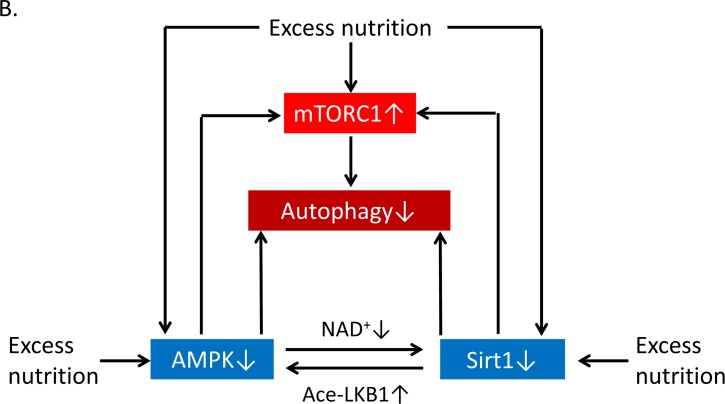

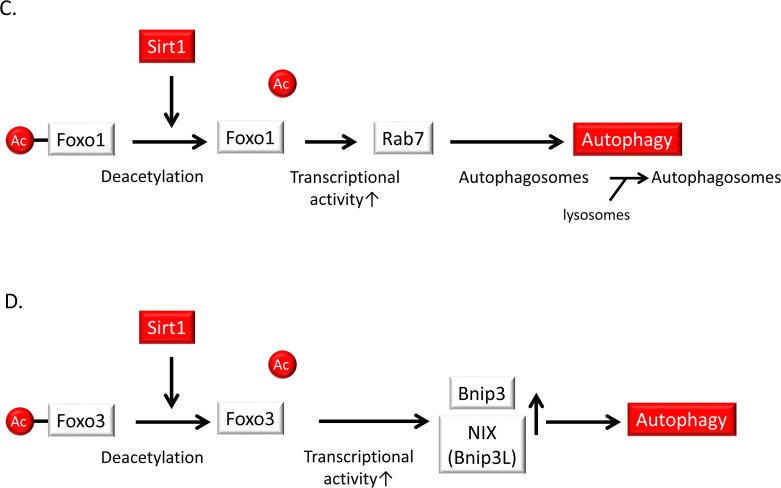

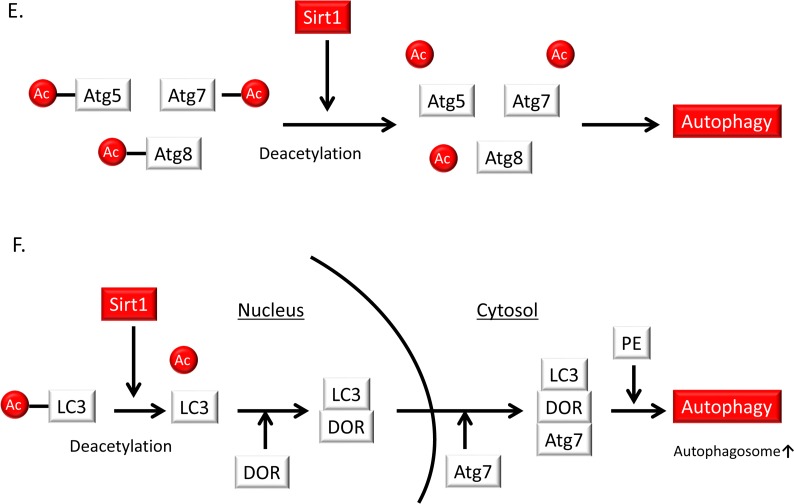
Relationship of autophagy levels to vascular aging and atherosclerosis and the role of Sirt1 in the regulation of autophagy (**A**) Basal autophagy and the mild adaptive autophagy response protect vascular cells against vascular aging and atherosclerosis through cellular homeostasis and protective degradation. By contrast, impaired or excessive autophagy leads to senescence, reduced stress resistance, oxidative stress, inflammation or cellular death, resulting in vascular aging and atherosclerosis. (**B**)Excess nutrition decreases autophagy initiation via mammalian target of rapamycin complex 1 (mTORC1) activation and reduced function of AMP-activated kinase (AMPK) and Sirt1. Nutrient-sensing pathways that include mTORC1, AMPK and Sirt1 engage in crosstalk with each other through changing of NAD^+^ level or acetylation/deacetylation of liver kinase B1 (LKB1). (**C**) Sirt1 induces the deacetylation, activation and nuclear translocation of FOXO1. FOXO1 activation stimulates the expression of Rab7, leading to the maturation of autophagosomes and autolysosomes via their fusion with lysosomes. (**D**) Sirt1 activation deacetylates and activates the FOXO3 transcription factor leading to subsequent Bcl-2/adenovirus E1B-19kDa interacting protein3 (Bnip3) or NIX/Bnip3-Like (Bnip3L)-mediated autophagy. (**E**) Sirt1 can form a molecular complex with several essential components of the autophagy machinery, including the autophagy-related genes (Atg) 5, Atg7 and Atg8. These autophagy components can be directly deacetylated by Sirt1. (**F**) Deacetylation of a nuclear pool of microtubule-associated protein light chain 3 (LC3) by Sirt1 initiates autophagy. Sirt1 deacetylates LC3 during starvation conditions. Deacetylated LC3 interacts with the nuclear protein DOR, and both proteins relocate from the nucleus to autophagosomes in the cytoplasm. Deacetylated LC3 interacts with Atg7 and other components of the ubiquitin-like conjugation machinery, leading to LC3 conjugation onto phosphatidylethanolamine (PE) and its incorporation into the early autophagosomal membrane.

Autophagy is the only mechanism able to degrade large molecules, organelles, proteins, and end products; basal homeostatic autophagy eliminates damaged components and plays an important housekeeping role [79]. Impairment of autophagy is closely related to vascular aging, and maintaining appropriate autophagy activities may exert an anti-aging effect. An analysis of cultured endothelial cells from old versus young patients revealed that older endothelial cells showed higher levels of p62 compared to younger cells [80]. Basal autophagy can protect vascular tissue against oxidative stress or inflammation by degrading damaged organelles and proteins to promote cell survival (Figure 6A). Mild cellular stresses including oxidative stress, oxLDL, advanced glycation end products (AGEs) and Endoplasmic reticulum (ER) stress also stimulate mild adaptive autophagy in vascular cells such as endothelial cells, VSMCs and macrophages; this autophagy protects against vascular injury (Figure 6A). Torisu et al. showed that atherosclerotic lesions were markedly increased in high-fat diet-fed ApoE^−/−^ autophagy-related gene (Atg) 7^endo^ (a conditional deletion Atg7 within endothelium) mice compared with in ApoE^−/−^ control mice, indicating that endothelial autophagy is critical for limiting lipid accumulation within the vessel wall [81]. In addition, oxidative stress or inflammation coupled with insufficient autophagy by macrophages may play a crucial role in the development of atherosclerotic plaques. Previous studies have shown that macrophage autophagy is implicated in the pathogenesis of atherosclerosis, and deficiencies in macrophage autophagy promote vascular inflammation, oxidative stress, and plaque necrosis via hyperactivation of the inflammasome and elevated interleukin 1 beta (IL-1β) production [82] [83]. Additionally, lipophagy may play a role in the hydrolysis of stored cholesterol droplets in macrophages, thereby facilitating cholesterol efflux and reducing atherosclerotic lipid accumulation [84]. Autophagy is also activated in VSMCs in vascular disease, which is important for VSMC survival and plasticity [85]. Grootaert et al. demonstrated that defective autophagy in VSMCs promotes senescence, ligation-induced neointima formation and diet-induced atherogenesis and prevents oxidative stress-induced cell death [86]. Thus, the impairment of autophagy likely contributes to the pathogenesis of atherosclerosis. However, severe oxidative stress or inflammation stimulates excessive autophagy in smooth muscle cells, endothelial cells and macrophages, leading to autophagic cell death, reduced collagen synthesis, fibrous cap thinning, plaque destabilization, lesional thrombosis, restenosis and acute coronary events [87,88] (Figure 6A). Thus, basal autophagy and a mild adaptive autophagy response may exert protective effects against cellular stresses such as inflammation and oxidative stress, leading to delays in vascular aging and age-related diseases such as atherosclerosis (Figure 6A). Therefore, the regulation of autophagy in the vascular wall may be a therapeutic target for the prevention of vascular aging and atherosclerosis.

Excess nutrition is implicated in metabolic dysfunctions such as insulin resistance, obesity, diabetes and dyslipidemia, all of which accelerate vascular aging and atherosclerosis; these conditions are associated with impaired autophagy. During excess nutrient conditions, alteration of nutrient-sensing pathways such as mammalian target of rapamycin complex 1 (mTORC1) activation and impaired AMP-activated kinase (AMPK) and Sirt1 activity leads to the suppression of autophagy. Previous reports have shown that excess nutrients such as high glucose concentrations or palmitate impair AMPK activity [89, 90, 91] and Sirt1 activity [66, 92, 93, 94] as well as mTORC1 activation in many cells including endothelial cells (Figure 6B). The alteration of nutrient-sensing pathways under excess nutrition conditions leads to the suppression of autophagy [95] (Figure 6B). When focusing on the role of Sirt1 in the regulation of autophagy, Sirt1 may activate autophagy by deacetylating FOXO1 and FOXO3 [96, 97, 98, 99] in the nucleus (Figure 6C and D). In the cytosol, Sirt1 targets Atg5, Atg7, and Atg8 (which are autophagy proteins required for the formation and elongation of the autophagosomal membrane) as substrates of deacety-lation [100, 101] (Figure 6E). Huang et al. also demonstrated that Sirt1 positively regulates starvation-induced autophagy by promoting Microtubule-associated protein light chain 3 (LC3) nucleocytoplasmic transport through the deacetylation of LC3 [102] (Figure 6F). Additionally, in mouse embryonic fibroblasts, overexpression of wild-type Sirt1 but not deacetylase-deficient Sirt1 increased basal autophagy [101]. Thus, Sirt1 can regulate autophagy by direct modulation via deacetylation of autophagy-related molecules. By contrast, the nutrient-sensing pathways including AMPK, Sirt1 and mTORC1 engage in crosstalk with each other. [46, 103, 104, 105, 106, 107, 108]. AMPK activates Sirt1 by increasing NAD^+^ production, and Sirt1 can conversely activate AMPK through LKB1 deacetylation and its activation [105, 106] (Figure 6B). Additionally, Sirt1 suppresses mTORC1 by interacting with tuberous Sclerosis Complex 2 (TSC2) [107], which is a negative regulator of mTORC1 (Figure 6B). We also reported that inhibition of Sirt1 in human THP-1 cells impaired autophagy and was associated with mTORC1 activation and reduced AMPK activation [108]. Thus, Sirt1 positively regulates autophagy via interactions with mTORC1 and AMPK in response to nutrient conditions. However, it is still unclear whether Sirt1 in endothelial cells and VSMCs regulates autophagy and protects against atherosclerosis. Therefore, further studies are necessary to elucidate this point.

## CONCLUSION

Sirt1 in endothelial cells, monocytes/macrophages and VSMCs protects vascular tissue by conferring resistance to cellular stresses (e.g., oxidative stress and inflam-mation) and senescence. Additionally, autophagy in endothelial cells, monocytes/macrophages and VSMCs could play a role in the pathogenesis of vascular aging and atherosclerosis. Sirt1 is implicated in the regulation of autophagy through the modulation of autophagy-related molecules and the interactions with other nutrient-sensing molecules including mTORC1 and AMPK, which play a central role in the regulation of metabolism and aging. Therefore, the activation of Sirt1 in endothelial cells, monocytes/macrophages and VSMCs should be considered as a novel therapeutic target to prevent atherosclerosis associated with vascular aging.
